# On the robustness of generalization of drug–drug interaction models

**DOI:** 10.1186/s12859-021-04398-9

**Published:** 2021-10-04

**Authors:** Rogia Kpanou, Mazid Abiodoun Osseni, Prudencio Tossou, Francois Laviolette, Jacques Corbeil

**Affiliations:** 1 Computer Science and Software Engineering, Université Laval, 1065, av. de la Médecine, Quebec, CA Canada; 2 InVivo AI, Mila - 180 Corporate Lab L, 6650, 01 Rue Saint-Urbain, Montreal, CA H2S 3G9 Canada; 3 Department of Molecular Medicine, Université Laval, 1065, av. de la Médecine, Quebec, CA Canada

**Keywords:** Drug–drug interaction, Side effects, Deep learning, Robustness, Generalizability

## Abstract

**Background:**

Deep learning methods are a proven commodity in many fields and endeavors. One of these endeavors is predicting the presence of adverse drug–drug interactions (DDIs). The models generated can predict, with reasonable accuracy, the phenotypes arising from the drug interactions using their molecular structures. Nevertheless, this task requires improvement to be truly useful. Given the complexity of the predictive task, an extensive benchmarking on structure-based models for DDIs prediction was performed to evaluate their drawbacks and advantages.

**Results:**

We rigorously tested various structure-based models that predict drug interactions using different splitting strategies to simulate different real-world scenarios. In addition to the effects of different training and testing setups on the robustness and generalizability of the models, we then explore the contribution of traditional approaches such as multitask learning and data augmentation.

**Conclusion:**

Structure-based models tend to generalize poorly to unseen drugs despite their ability to identify new DDIs among drugs seen during training accurately. Indeed, they efficiently propagate information between known drugs and could be valuable for discovering new DDIs in a database. However, these models will most probably fail when exposed to unknown drugs. While multitask learning does not help in our case to solve the problem, the use of data augmentation does at least mitigate it. Therefore, researchers must be cautious of the bias of the random evaluation scheme, especially if their goal is to discover new DDIs.

**Supplementary Information:**

The online version contains supplementary material available at 10.1186/s12859-021-04398-9.

## Background

In modern pharmacotherapy, it is common to use drug cocktails to deal with one or multiple diseases affecting an individual. Unfortunately, some of these therapies result in drug–drug interactions (DDIs) when two or more drugs interact and alter each other expected behavior (i.e. pharmacokinetic (PK), pharmacodynamic (PD), absorption, distribution, metabolism, excretion, efficacy, or toxicity) [1]. Generally, DDIs can be avoided. However, if undetected before administration, they can harm patients and incur substantial costs to the health care systems.

Traditionally, in vivo experiments and clinical trials were used to detect and characterize DDIs [2, 3]. However, due to the vast number of multi-drug therapies and the large amount of labor, time, and costs required by these procedures, systematic screening of all drug combinations is unrealistic. Moreover, due to the cohort size in clinical trials, many DDIs remain undetected or mischaracterized until drugs are approved and treatments are administered to patients [4]. It creates a substantial workload for post-approval and drug safety surveillance agencies. These agencies must be alert all time to detect and prevent the administration of harmful drug combinations.

Over the last decade, in silico methods have shown the capability to detect and characterize certain DDIs [5–28]. These methods are inexpensive and fast compared to evidence-based methods. Despite being less accurate than evidence-based approaches, in silico methods have truly assisted with the current understanding of DDIs and consequently with therapy recommendations and prioritization [29]. These methods can be roughly classified into three categories : text mining-based, machine learning-based, and deep learning-based methods [8, 17–22, 25–28]. Text mining-based methods allowed to extract already annotated and known DDIs from the literature, while machine and deep learning-based methods are a promising way to identify and characterize non-annotated potential DDIs. The accumulation of data from the scientific literature and electronic medical records has strongly favored the development of the field [30, 31].

Nevertheless, in silico computational methods warrant additional investigation and validation before being fully adopted and integrated into pharmacotherapy development and regulatory processes. This reluctance is primarily due to the lack of comprehension and trust in the robustness and the readiness of DDIs modeling for real-world applications. The absence of training, evaluation, and model deployment consensus also deters the integration. Many studies report performance metrics that can be hardly attributed to modeling improvements alone [8, 17–22, 25–28].

The DDIs prediction models’ performances are currently predicated on the data partitioning process, i.e., training and testing sets. The need to standardize this process is urgent since the outcomes of these models will differ depending on the intended use and how the modeling was performed. In addition, most DDIs models’ performance reports do not reflect their true generalization capabilities. Indeed, studies usually report aggregated performance over many types of DDIs, giving an unrealistic view of the capabilities and limitations of the models. This performance misrepresentation is due to the unequal distribution of DDIs in the population. Since the accuracy of deep learning models is dependent on the data size, reporting aggregated results thus hides these nuances and pushes the field towards improving average performance while neglecting the worst-case performance. Therefore, a detailed performance report is necessary to improve DDIs prediction where it matters the most with a finer and in-depth comparison of modeling approaches. Another major problem is the lack of consensus on the input information needed for accurate DDIs modeling [32]. Most of the available models often use different combinations of input features among molecular structure, target information, pathways, individual drug side-effects, Absorption–Distribution–Metabolism–Excretion–Toxicity (ADMET) profiles, target indications, among others [20–24]. Thus, the deployment environment for DDIs prediction models depends on the availability of these features at the different drug development stages. For example, using molecular structures alone allows DDIs prediction models to be used at any stage of the drug discovery process, while using ADMET profile limits models to late stages only. In comparison to methods using different feature combinations, [17, 19, 28, 32] reported a state-of-the-art performance by simply improving molecular representation and using molecular structures. Therefore, enhancing molecular representation should be the common ground for improved DDIs prediction models favoring their deployment at any drug and therapy development stage. However, the lack of standardization and accepted procedures creates confusion about what constitutes state-of-the-art DDIs prediction models.

This study investigates the robustness of the generalization of molecular structure-based DDIs prediction models. The motivation is the unrestricted deployment of such models, for example, in the early stages of the drugs discovery process, where they could assist candidate prioritization, improve downstream attrition in clinical trials, and aid in understanding the mechanism of action of some candidates. Our investigation evaluated the potential of structure-based DDIs models to be used in an unrestricted fashion in drug development and identified some of the hurdles impeding this goal. Hence, we tested a three-level scenario the capability of current methods to generalize and define the applicable parameters. We also investigated how to remove some of the limitations to maximize the use of existing models to provide practical applications in the field.

The paper is structured as follows. In “Problem formalism” section, we present the DDIs problem and clarify any assumptions that were made and set the expectations about the results and the methods. “Evaluation schemes” section presents three evaluation schemes that test different levels of generalization, which will highlight the capabilities of current DDIs prediction models. In the “Modelling” and “Featurization” sections, a general multi-label classification engine is presented. It generalizes previous structure-based DDIs models by using state-of-the-art feature extraction techniques. “Data and partitioning” and “Metrics” sections provide details about the datasets and the metrics which were used to train, evaluate and assess models’ performances. Finally, the “Results” section presents the experiments and analyzes the current level of generalization that can be achieved with structure-based models. It also discusses the factors that impact this ability to generalize and discover new DDIs using imperfect models.

## Material and methods

### Problem formalism

Let $x := (x^{1}, x^{2})$ be a drug pair, with $x^{1}$ and $x^{2}$ being two drugs belonging to a molecular space ${{\mathcal {X}}}$. The associated phenotypes form a set denoted $y := \left\{ y^{1}, \ldots , y^{n} \right\}$, where $y^{j}$ belongs to a set ${{\mathcal {Y}}}$ of *n* predefined phenotypes. Given a database *D*, containing *m* drug pairs with associated phenotypes (either side effects or PK/PD effects), DDIs characterization models aim to predict *y* given a pair *x*. It is often assumed that having *x* associated with *y* means that no other phenotypes in ${\overline{{{\mathbf {y}}}}} := {{\mathcal {Y}}}{\setminus } {{\mathbf {y}}}$ associated with *x*. This assumption is relatively strong, as it is likely that some phenotypes in ${\overline{{{\mathbf {y}}}}}$ that are associated with *x* have not yet been observed or discovered. The probability of such events depends on the quality and the exhaustiveness of the experiments conducted to build *D*. Considering this assumption, DDIs characterization is a task with noisy and imbalanced datasets. The level of imbalance grows with $\left|{{\mathcal {Y}}}\right|$ when most drug pairs have only a few associated phenotypes (i.e. $\left|{{\mathbf {y}}}\right| \ll \left|{{\mathcal {Y}}}\right|$ as is often the case in practice (see Table 1). Another factor contributing to the imbalance is the uneven frequency distribution of phenotypes in ${{\mathcal {Y}}}$. As the severity of a phenotype is often inversely correlated to its frequency, *D* will often have phenotypes whose frequencies are significantly unequal (see DrugBank database in Fig. 2, where 93% of reported phenotypes appear only in 5% of the dataset). This formalism and its assumptions may seem obvious, but they remain significant discrepancy sources between studies. Experimental design and model assessment of different studies must account for them to enable a fair comparison and faithfulness to reality. We detail in “Evaluation schemes” and “Metrics” sections our experimental design and procedures that derive from this formalism.

**Fig. 1:**
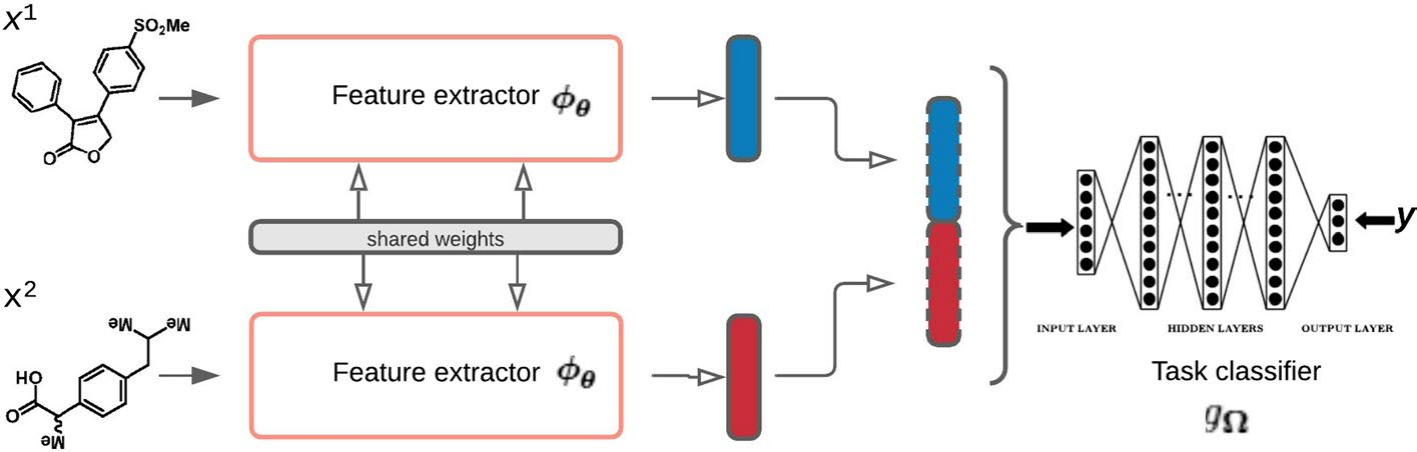
Overall framework. The main steps are as follows. First, a feature extractor network to map inputs $x := (x^{1}, x^{2}) \in {{\mathcal {X}}}$ to a latent space. Then, each pair of drugs is represented as a feature vector by concatenating the corresponding latent features of the drugs. Last, the feature vectors representing the drug pairs are fed into a deep neural network to train the predictor to uncover potential DDIs

### Modelling

To lead our investigation of molecular structure-based methods, we present a general deep learning architecture (Fig. 1) that encapsulates recent structure-based DDIs models [17, 18, 28, 32–35]. Given the formalism presented, DDIs characterization is best viewed as a multilabel classification task to take advantage of the correlations between phenotypes. The goal is to produce a function that maps each input ${{\mathbf {x}}}$ to a binary vector ${{\mathbf {b}}}\in {\mathbb {B}}^{\left|{{\mathcal {Y}}}\right|}$ (with ${\mathbb {B}} := \left\{ 0, 1 \right\}$). Each position *j* of that binary vector corresponds to a phenotype $y^j \in {{\mathcal {Y}}}$ such that ${{\mathbf {b}}}^{j} := 1$ if $y^j \in {{\mathbf {y}}}$ and 0 otherwise. Consequently, existing methods that fit into this view produce DDIs models *f* having the following form:

$$\begin{aligned}&f : {{\mathcal {X}}}\times {{\mathcal {X}}}\rightarrow {\mathbb {B}}^{\left|{{\mathcal {Y}}}\right|} \\&{{\mathbf {x}}}= (x^1, x^2)\mapsto g_{{\varvec{\Omega }}}\left( \left[ {\varvec{\phi }}_{{\varvec{\theta }}}(x^1), {\varvec{\phi }}_{{\varvec{\theta }}}(x^2) \right] \right) , \end{aligned}$$

where ${\varvec{\phi }}_{{\varvec{\theta }}}$ is a molecular feature extractor of parameters ${\varvec{\theta }}$, $g_{{\varvec{\Omega }}}$ is a fully connected network of parameters ${\varvec{\Omega }}$, and $\left[ \ldots \right]$ is the concatenation operator.

All hidden layers of the network $g_{{\varvec{\Omega }}}$ have the same number of units (selected by cross-validation) and use the *ReLU* activation function. The output layer has $\left|{{\mathcal {Y}}}\right|$ units and uses the *Sigmoid* activation function, meaning that $g_{{\varvec{\Omega }}}$ output for each $y^{j}$ in ${{\mathcal {Y}}}$, the probability that it could be induced by the interaction between $x_1$ and $x_2$. These probabilities can be converted into binary outputs using a standard threshold or different thresholds per phenotype. We will discuss in the “Metrics” section how to handle these thresholds at evaluation. The whole network *f* is trained end-to-end to find the parameters ${\varvec{\Omega }}$ and ${\varvec{\theta }}$ that optimize the following objective (multilabel binary cross-entropy):

1 $$\begin{aligned} \underset{{\varvec{\Omega }}, {\varvec{\theta }}}{\mathrm {argmin}}\ { \underset{({{\mathbf {x}}}, {{\mathbf {y}}})}{{{{\mathbf {E}}}}}\ { -\left[ \sum _{\begin{array}{c} y_k \in {{\mathbf {y}}} \end{array}} {{\mathbf {b}}}_k \log {\hat{{{\mathbf {b}}}}}_k + \sum _{\begin{array}{c} y_k \in {\overline{{{\mathbf {y}}}}} \end{array}} (1 - {{\mathbf {b}}}_k) \log (1 - {\hat{{{\mathbf {b}}}}}_k)\right] },} \end{aligned}$$

where ${\hat{{{\mathbf {b}}}}}:= f({{\mathbf {x}}})$. To summarize, given a drug pair ${{\mathbf {x}}}$, *f* first computes the representation of each of its drugs $(x_1, x_2)$. After that, it concatenates the obtained vectors. Finally, this concatenation is passed to a non-linear network $g_{{\varvec{\Omega }}}$ to get the probability of each phenotype associated with that pair. Using a threshold, it is then straightforward to infer all the phenotypes associated with the pair.

### Featurization

**Fig. 2:**
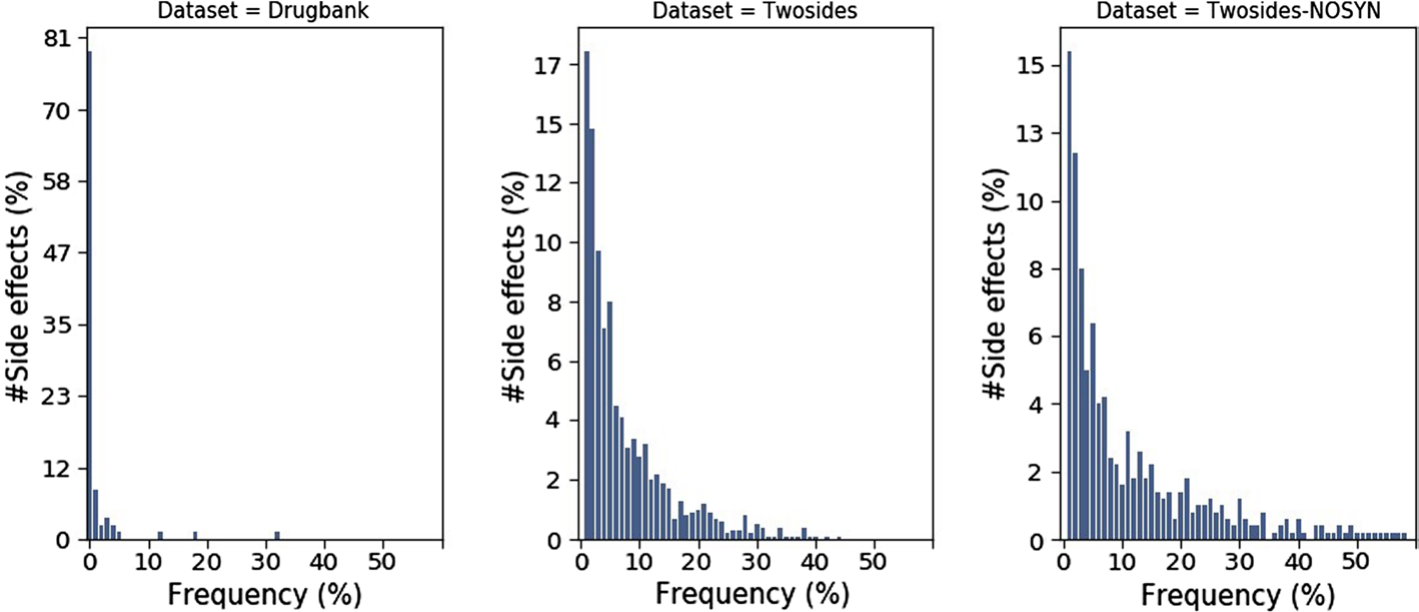
Distribution of phenotypes frequencies in DrugBank (left), Twosides (center), and Twosides-NOSYN (right)

The feature extractor ${\varvec{\phi }}_{{\varvec{\theta }}}$ is the main difference between existing molecular structure-based DDIs prediction models. Herein, we explore various choices of molecular representation and feature extractors. Specifically, we use three of the most popular molecular representations: ECFP6 (Extended Connectivity FingerPrints of diameter 6), SMILES (Simplified Molecular-Input Line-Entry System), and molecular graphs (Fig. 2).

*ECFP6* [36] are binary vectors encoding the environment around each atom of a molecule. The surroundings are delimited by the radius going from 0 to 3. These fingerprint vectors have 2048 bits and are obtained using the RDKit package [37]. Following [17], these fingerprints were first transformed into structural similarity profiles (SSP). Next, higher-level molecular features were extracted using the feature extractor ${\varvec{\phi }}_{{\varvec{\theta }}}$. The latter takes the form of a fully connected network with many ReLU and batch normalized activated hidden layers. The exact number of layers and units is selected by grid search. Following [17], we will denote the entire model using this combination of fingerprints and network as DeepDDI.

*SMILES* [38] are string-based structural representations of chemical compounds. Each character is first embedded into a continuous space using an embedding layer to extract molecular features from a string representation. Next, a Bidirectional Long Short Term Memory (BLSTM) [39] or a one-dimensional Convolutional Neural Network (1D-CNN) [40] architecture processes the sequence of embeddings representing the SMILES. For the BLSTM, multiple ReLU activated hidden layers are used. The output of the network of the SMILES last character at the last layer is the output of the feature extractor. For the 1D-CNN, there are also multiple ReLU-activated hidden convolution layers. Each layer has kernels of size 7 (i.e. they span seven characters of the input sequence) and is followed by batch normalization and max-pooling over windows of size 2 before the next convolution. At the final convolution layer, max-pooling is performed over the whole sequence to have a representation that summarizes the entire sequence. Together, the embedding layer with the BLSTM or 1D-CNN networks represents the feature extractors for SMILES. The number of BLSTM/1D-CNN layers and the number of units per layer are all selected by a grid search. We will later use *BLSTM* and *CNN* to refer to the networks using SMILES with BLSTM and 1D-CNN as feature extractors.

*Molecular graphs* are among the most natural methods to represent molecular structures, as atoms and bonds in a molecule can be mapped directly to nodes and edges in a graph. To extract features from molecular graphs, we define ${\varvec{\phi }}_{{\varvec{\theta }}}$ as a graph convolutional network (for a comprehensive review on GCNs, please see [41]). More precisely, we use the Graph Isomorphism Network (GIN) architecture [42] using two different pooling strategies. GIN has been theoretically proven to have the maximum discriminative power among GNNs. GIN can map isomorphic graphs (i.e. topologically similar graphs) to the same representation and non-isomorphic ones to different representations. Specifically, GIN uses a multi-layer perceptron (MLP) model to update the node features [42]. In the first, no pooling is performed after each internal GIN layer, but the average-pooling is done after the last layer to compute the extractor output. In the second, Laplacian pooling (LaPool) [43] hierarchically captures the molecule’s structure at each hidden layer before applying a sum-pooling at the last layer. Lastly, LaPool iteratively coarsens (collapses nodes to single one) graphs to allow for message passing updates to reach distant nodes in the original graph. The main intuition is to measure a node-level smoothness to determine which nodes to use as pooling centers. The smoothness indicates the difference in signal between each node and its neighbors. We consider these two architectural variations because the GCNs pooling layers can significantly impact generalization. We will later refer to the models using these feature extractors as GIN and GIN + LaPool.

### Data and partitioning

We trained and evaluated our DDIs prediction models using **DrugBank** and two versions of **Twosides**. These datasets have unique characteristics that allow us to draw more robust conclusions from our experiments.

DrugBank provides a repository of experimentally discovered PK/PD interactions and clinically validated side effects. It has been introduced by [17] using Drugbank v5.0.3. It has been preprocessed to retain phenotypes that appear at least five times in the database (the preprocessed version of the database is available at repository). Overall, it covers 86 types of polypharmacy side effects over 192,284 unique drug pairs.

Twosides is a database mined from text sources. It contains unsafe co-drug prescriptions reported by clinicians after drug approval. It was introduced by [44] and is freely accessible on the PharmGKB website. Initially, it comprised 1318 unique side effects over 63,473 drug pairs. By removing all phenotypes that appear fewer than 500 times in the dataset, we reduced the size to the 964 most frequent phenotypes. This version of the database is referred to as Twosides.

Zhang et al. [45] has previously reported that many side effects from the Twosides database are semantically and physiologically similar. For example, *abnormal blood pressure*, *high blood pressure*, and *increased blood pressure* relate to the same side effect. Similarly, the side effect of *blood disorder* is related to *anemia and hemorrhage*, while *hypocalcemia* is synonymous with *decreased blood calcium*. Other side effects such as *alcohol abuse* or *drug abuse* are linked to non-physiological factors and cannot be considered *pure* side effects. As a result, following [45], we removed specific side effects and group the remaining using the Unified Medical Language System (UMLS)—Metathesaurus. UMLS has been developed and maintained by the United States National Library of Medicine (NLM). Its Metathesaurus is one of the most comprehensive terminological systems in biomedicine [46]. It is organized by concept and links similar phenotypes from nearly 200 different lexicons to the same idea. It also identifies valuable relationships between concepts and preserves the meanings and associations from each lexicon. We downloaded the associations for each of the 964 side effects and filtered them to keep only those including side effects belonging to our dataset. After synonym clustering, the set of phenotypes was reduced to 477 side effects. We refer to this smoothed version of the Twosides database as Twosides-NOSYN. Table 1 summarizes the characteristics of the datasets, namely the number of pairs, drugs, phenotypes, and the label density (LD), which refers to the ratio of positive and negative values in the dataset.

**Table 1:** Statistics of the data sources

Database	*m*	$\left|{{\mathcal {Y}}}\right|$	Nb of unique drugs	Median of $\left|{{\mathbf {y}}}\right|$	LD
Twosides	63,472	964	645	72.126	0.075
Twosides-NOSYN	63,472	477	645	77.61	0.16
DrugBank	191,878	86	1710	1.002	0.012

### Evaluation schemes

A common practice in machine learning is to divide the dataset into training, validation, and testing partitions. A learning algorithm uses the training partition to produce a model. The validation partition is then used to select the best model between those learned with different hyperparameters combinations at the training phase. To avoid overfitting, we apply the early stopping [47] method at the training phase. Finally, the testing partition is used to evaluate the model’s performance on data not seen during both training and validation. Typical split percentages are 80% for training and 20% for testing. The generalization guarantees associated with any machine learning model can be provided by using the testing set performances if this set comes from the same data distribution as the training set [48, 49]. However, for these guarantees to hold in practice, the testing set should also match the data distribution that will be seen after the model is deployed. Therefore, it is critical to consider how to construct training and evaluation sets from our database *D* that have statistically solid guarantees when deploying our DDIs models prospectively.

Randomly splitting the data is often viewed as the best practice for a strong guarantee at test time. Still, it is worth considering the implications for DDIs prediction and characterization (also bootstrapping, i.e. redo the random split many times). Specifically, randomly splitting drug pairs is not the same as randomly splitting the set of drugs that have generated all these pairs. These two splitting procedures will give very different generalization performances and guarantees on the final model. For the remainder of this section, we explore three splitting schemes used for DDIs characterization and comment on their application to real-life use.

#### Random split

In automatic discovery and annotation of DDIs, a model is intended to discover and characterize DDIs among drugs in a database *D*. In such cases, after deployment, the model will only be exposed to drugs that have been seen during training even if the pairs that the model is asked about are unseen (i.e. $x:=(x^{1}, x^{2})$) is unseen in training but $x^{1}$ and $x^{2}$ have been seen with other drugs). Consequently, randomly splitting the drug pairs into training, validation, and testing sets is the appropriate evaluation scheme because the generalization on unseen pairs in the database (not unseen drugs) is the goal.

#### One-unseen split

When the model is meant to discover and characterize DDIs between new drugs and those that already exist in *D*, it is critical to refrain from randomly splitting the data. Instead, the splitting must be done so that for each pair seen during validation, only one of its drugs has been seen during training. Likewise, only one of its drugs must be seen during training or validation for each pair in the testing partition. Failing to do so will make the deployment settings different from the validation and testing settings, ultimately leading to over-confidence (overfitting) in the model’s capabilities. This scenario is relevant when using our DDIs models to predict safety liabilities associated with taking recently approved drugs in combination with those that already exist.

#### Both-unseen split

**Fig. 3:**
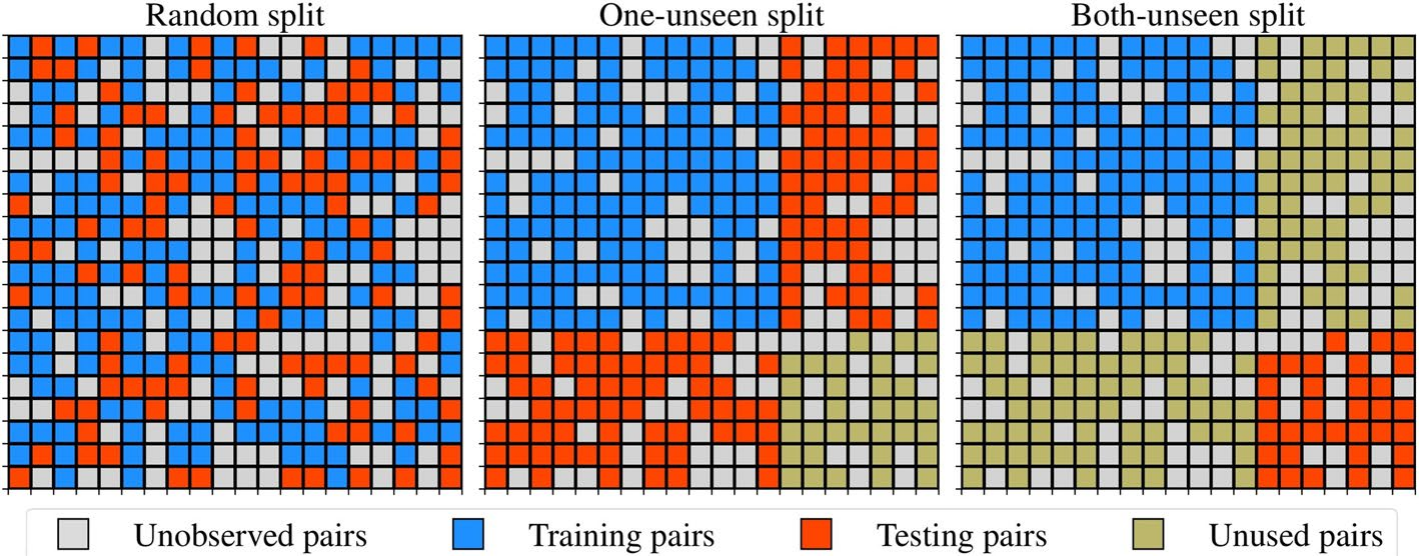
Evaluation schemes for DDIs models. Left: the random splitting strategy, center: the one-unseen splitting strategy, Right: the both-unseen splitting strategy. It is recommended to combine the strategy at the center and the right to avoid unnecessarily wasting data. One-unseen and both-unseen share the same training examples

Moreover, we may be interested to know at some point how safe it is to combine two newly approved drugs using our existing DDIs models. Alternatively, we may want to take advantage of existing DDIs models when exploring new drug combinations. In this case, the goal is to predict how two drugs that have never been seen during the training might interact with one another. Therefore, the database must be split so that the validation and testing partitions contain only pairs for which both drugs are unseen during training. Gaining insight into the performance under this scenario, it is critical to understand if our models currently understand the molecular mechanisms behind DDIs or simply memorize patterns in the data seen during training. Figure 3 summarizes all three evaluation schemes. The training partition is the same in *one-unseen* and *both-unseen* scenarios, while their sets of unused and testing pairs are mutually exclusive. Consequently, to compute performance in both schemes, one can train and then compute performance on the unused and testing pairs of one scenario separately. This helps to avoid doubling the training time unnecessarily.

### Metrics

There are various classification metrics suitable for the multilabel classification task, namely: Precision, Recall, F1-score, AUROC, and AUPRC. However, due to the labels imbalance in the datasets used, we favored using AUPRC (area under the precision-recall curve) instead of reporting the results only in AUROC (area under the receiver operating characteristic curve) [50].

Recall that a ROC curve summarizes the trade-off between the true positive rate ($TPR := TP/(TP + FN)$) and the false positive rate ($FPR := FP/(FP + TN)$) for a model using different probability thresholds, where TP, TN, FP, and FN stand for True Positive, True Negative, False Positive, and False Negative. Using different probability thresholds, a precision-recall curve computes the trade-off between the true positive rate and the positive predictive value. The area under a precision-recall curve $AUPRC:= \sum _n (R_n - R_n-1) P_n$ is computed as the weighted mean of precision $P_n$ achieved at the *nth* threshold, with the increase in recall $(R_n - R_n-1)$ from the previous thresholds. Intuitively, it measures the classifier’s ability not to label a negative sample as positive under any given threshold.

## Results

### Investigating the importance of evaluation scheme

**Fig. 4:**
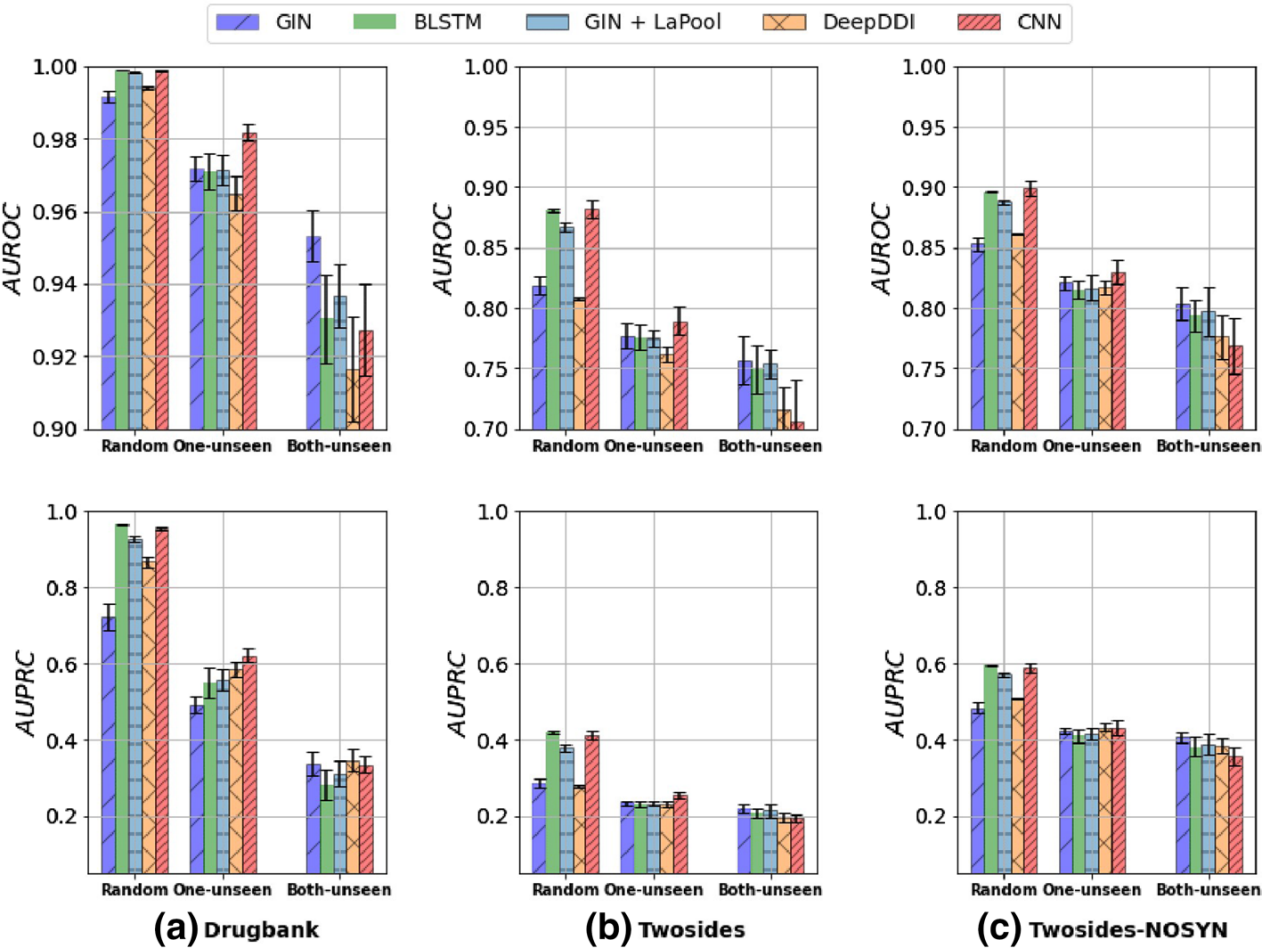
Models performances on random, one-unseen, and both-unseen evaluation schemes. The AUROC is given on top, and the AUPRC is shown at the bottom. The scores are presented with their mean standard deviation obtained through ten independent runs with random seeds. Again, axes are on different scales

One of the main assumptions is to evaluate the impact of the partitioning process on the models’ performances. We trained and evaluated on Drugbank, Twosides, and Twosides-NOSYN all the structure-based DDIs models presented in the “Featurization” section, namely 1D-CNN, BLSTM, GIN, GIN + LaPool, and DeepDDI. Each model is evaluated using every evaluation scheme (random split, one-unseen, and both-seen) across ten independent runs with different random seeds. Each run had different training, validation, and testing partitions. The dataset was divided into three (train/validation/test) sets for each run: 60/20/20 for DrugBank, 80/10/10 for Twosides, and Twosides-NOSYN, which are the smallest datasets. We report the average AUROC (top boxes) and AUPRC (bottom boxes) and their standard deviations on all runs in Fig. 4.

As we assume in “Problem formalism” section, that no other phenotypes in ${\overline{{{\mathbf {y}}}}}:= {{\mathcal {Y}}}\setminus {{\mathbf {y}}}$ associated with *x*, we trained our models using all ${\overline{{{\mathbf {y}}}}}$ as the negative samples.

Figure 4 shows that all model performances decrease significantly when the evaluation scheme changes from random to one-unseen to both-unseen for both of our datasets. However, the random split evaluation scheme models consistently outperformed. The decrease of the metrics indicates that the models are getting worse at predicting positive and negative labels. This implies that generalizing to new unseen drugs is difficult for all structure-based DDIs prediction methods.

To better understand the performance variation, we labeled each of the examples in the test set as follows: SS for a drug combination in the test set for which all the drugs have been seen in the training set; SU, a pair of drugs with a single one of them in the training set and UU a pair of drugs never seen in the training set. Table 2 presents the distribution of the SS, SU, and UU types in the test set for each evaluation scheme over the ten random runs. The random split evaluation scheme contains the most SS type pairs, and therefore the greatest number of drugs shared between the validation sets. This is what is expected since we randomly selected the drug pairs. On the other hand, one-unseen and both-unseen were the evaluation schemes with the least SS type pairs. Looking at the trend of the metrics and the distribution of the different drug pair types, we hypothesized that it is easier for models to propagate information from one drug seen to other drugs than from drugs never seen in training.

**Table 2:** Of drug combinations in the test set

Dataset	Splitting scheme	Nb. of drug combinations	SS	SU	UU
Drugbank	Random	38,363 ± 0	38,341 ± 5	21 ± 5	0 ± 1
	One-unseen	46,178 ± 1598	0 ± 0	46,178 ± 1598	0 ± 0
	Both-unseen	7580 ± 6834	0 ± 0	0 ± 0	7580 ± 6834
Twosides	Random	6348 ± 0	6347 ± 1	1 ± 1	0 ± 0
	One-unseen	9706 ± 630	0 ± 0	9706 ± 630	0 ± 0
	Both-unseen	629 ± 107	0 ± 0	0 ± 0	629 ± 107

The scores are presented with their mean standard deviation obtained through ten rounds of single training-test setups

### Investigating the impact of intra-class and inter-class imbalances

**Fig. 5:**
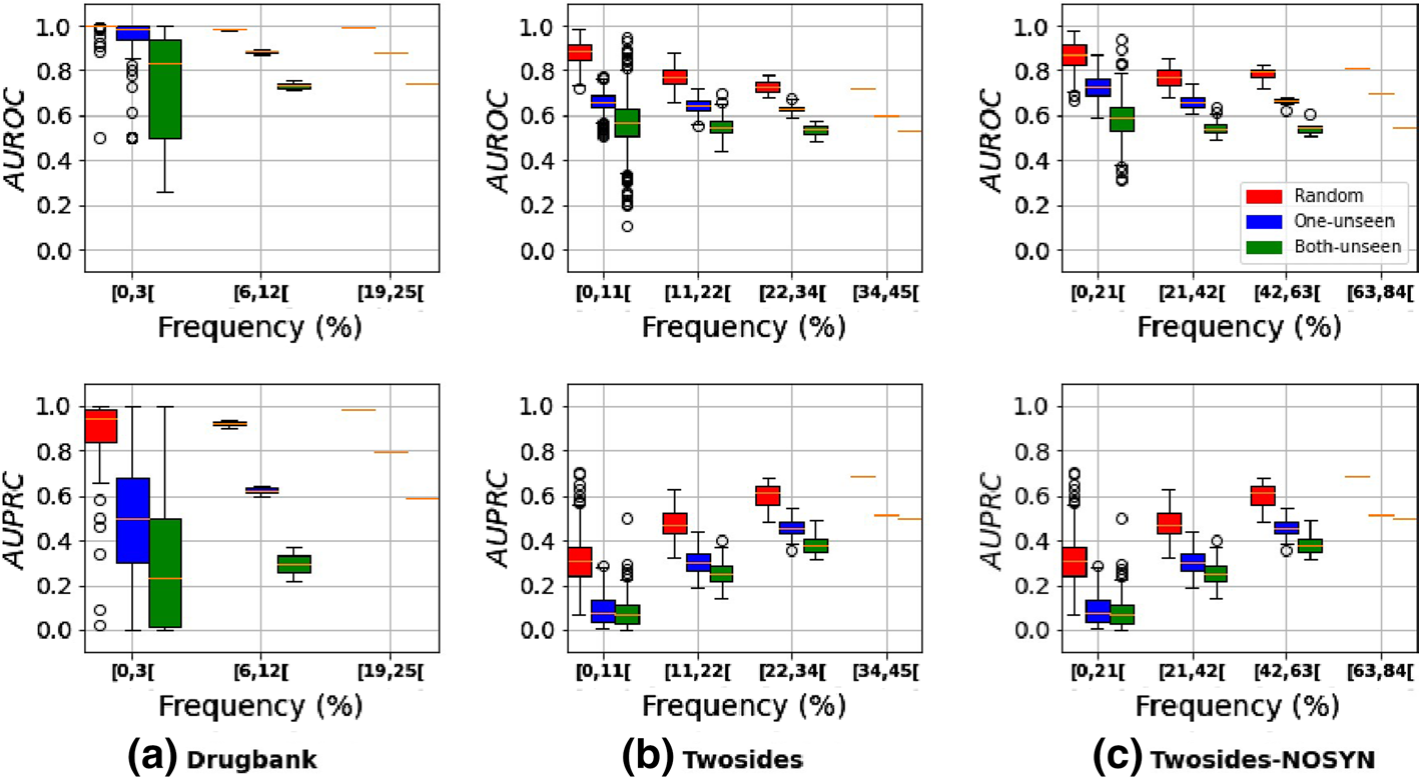
Impact of evaluation schemes on the different categories of phenotypes. The phenotypes are grouped according to their frequencies in the datasets. For example, [11,22[ in x-axis groups all phenotypes present at greater than 11% and less than 22% in the training set. On the other hand, for DrugBank, there are only 3 different intervals because there is no phenotype present above 25%, which is the case in Twosides and Twosides-NOSYN. AUPRC and AUROC scores are from our best model (1D-CNN) on the benchmark datasets

Generally, most of the DDIs discovery datasets are heavily imbalanced. There are two types of imbalances: the intra-class i.e. for the same phenotype, we have an imbalance between positive and negative samples. There is also the inter-class which means there is an imbalance representation overall between all the side effects to be predicted i.e. there are more examples of some side effects than others. The goal is to determine the impact of different proportions of positive and negative samples on the ability of the models to generalize well to new unseen drugs. To proceed, we measured the frequency of each phenotype in the test and the training sets. Then, we evaluated different bins (intervals) categories of phenotype frequency and obtained three bins for DrugBank and four bins for Twosides and Twosides-NOSYN (Fig. 5). Figure 5 shows the impact of the different evaluation schemes on the different phenotypes present in our datasets.

Like Fig. 4, Fig. 5 also confirms the trend of the more complex the evaluation scheme gets ((random < one-unseen < both-unseen)), the metrics (AUROC and AUPRC) decreased. But Fig. 5 also shows a similar behavior within the bins i.e. this decrease occurred consistently across all phenotypes no matter their frequencies. Therefore, the low-frequency phenotypes are affected by the difficulty of the evaluation scheme, i.e. the inter-class imbalance is equally affected no matter the phenotype or the dataset. Another observation was that the ratio of positive-to-negative samples for each phenotype correlates with the models’ ability to correctly label a sample under any threshold, suggesting that the intra-imbalance influences the learning process. For example, most phenotypes with low AUPRC ($\le$ 0.3) have several negative samples in the training set that were several times higher than the positive samples. As a result, only a few samples were predicted as positive, resulting in a low rate of true positives (TP) predictions. In contrast, side effects with a relatively high AUPRC ($\ge$ 0.8) and very low AUROC have more positive samples in the training set, and the ones with the most significant results (both AUROC and AUPRC high) are those with a ratio of negative/positive examples close to one. This highlights that when the number of positive examples for a phenotype grows, the models better distinguish positive and negative DDIs (unless positive DDIs outgrow the negative). Our results also suggest that the imbalanced nature of the task is an important impediment for learning and generalization regardless of the evaluation scheme. To overcome this hurdle, databases with more DDIs must be created, or better strategies to handle an imbalance in multilabel classification should be developed. In addition, using a single value of AUROC or AUPRC to report the DDIs model performances should be avoided as it is non-informative regarding the model predictive performance.

### Investigating the importance of drug encoding

The drug encoding step initiates the learning process. Since each model uses a specific drug representation, we evaluated the impact of the drug encoding on the learning process. Three types of representations were used: SMILES, fingerprints, and molecular graph representation. Figure 4 presents the results of all our encodings (1D-CNN BLSTM = SMILES, GIN & GIN + LaPool = molecular graph, DeepDDI = Fingerprint). It shows that overall performances vary between models. This expected behavior underscores the importance of using the right molecular representations with the right feature extractors.

Generally, the SMILES drug representation outperforms the fingerprint and molecular graph approaches in random and one-unseen evaluation schemes for AUPRC and AUROC (see Fig. 4). However, models using graph encoding such as GIN perform better in the most constraining evaluation scheme (i.e. both-unseen). Moreover, compared to the state-of-the-art, in the random scheme (the most common and used scheme in the literature), our model performances were on par or better than those reported in recent studies (Table 3).

**Table 3:** Performance comparison on Drugbank and Twosides datasets for the random split scheme

Dataset	Method	AUROC	AUPRC
Drugbank	DeepDDI [17]	0.994	0.866
	NDD [32]	0.954	0.922
	**1D-CNN (Ours)**	**0.999**	0.96
	SSI-DDI [28]	0.983	**0.981**
Twosides	MHCADDI [19]	0.882	–
	GENN [51]	0.886	0.260
	**1D-CNN (Ours)**	**0.889**	**0.420**

We hypothesize that the low predictive performance on Twosides arises from the low number of training examples, as Twosides is much smaller than DrugBank. Interestingly, these scores show that the general architecture used is as robust as any recently published random split models. Even those outside of the structure-based DDIs, such as adjacency matrix factorization (AMF) [33], who represent the problem as a link prediction problem, reported 0.991 AUROC and 0.950 AUPRC. Zitnik et al. [18] (Decagon), a GCN network over a multimodal graph of protein–protein interactions, drug–protein target interactions, and drug–drug interactions, reported an AUROC of 0.872. Shtar et al. [33] and Zitnik et al. [18] handle the data imbalance through random under-sampling from the set of negative samples at a ratio corresponding to the positive set. As mentioned above, all negative examples have been considered intentionally.

We also noticed that the drop in performance is not drastic for all the evaluated models. For example, compared to other models, the change of evaluation scheme does not significantly affect the graph network (GIN). Moreover, the difference in performance between recurrent networks (1D-CNN, BLSTM) and graph networks is much more significant when one is in the random mode.

### Investigating the robustness of models

Once the models are built, we accessed their robustness, i.e. how consistently accurate the output is even if one or more of the features of the examples in the dataset or the assumptions about those examples are drastically changed. This validation finds its legitimacy in the simple fact that a robust model (if we consider the different measured metrics) is not necessarily a powerful model. There are other criteria beyond metric performances which must be validated to assure the robustness of a model before its deployment, including its stability and sensitivity (tolerance to noise).

**Fig. 6:**
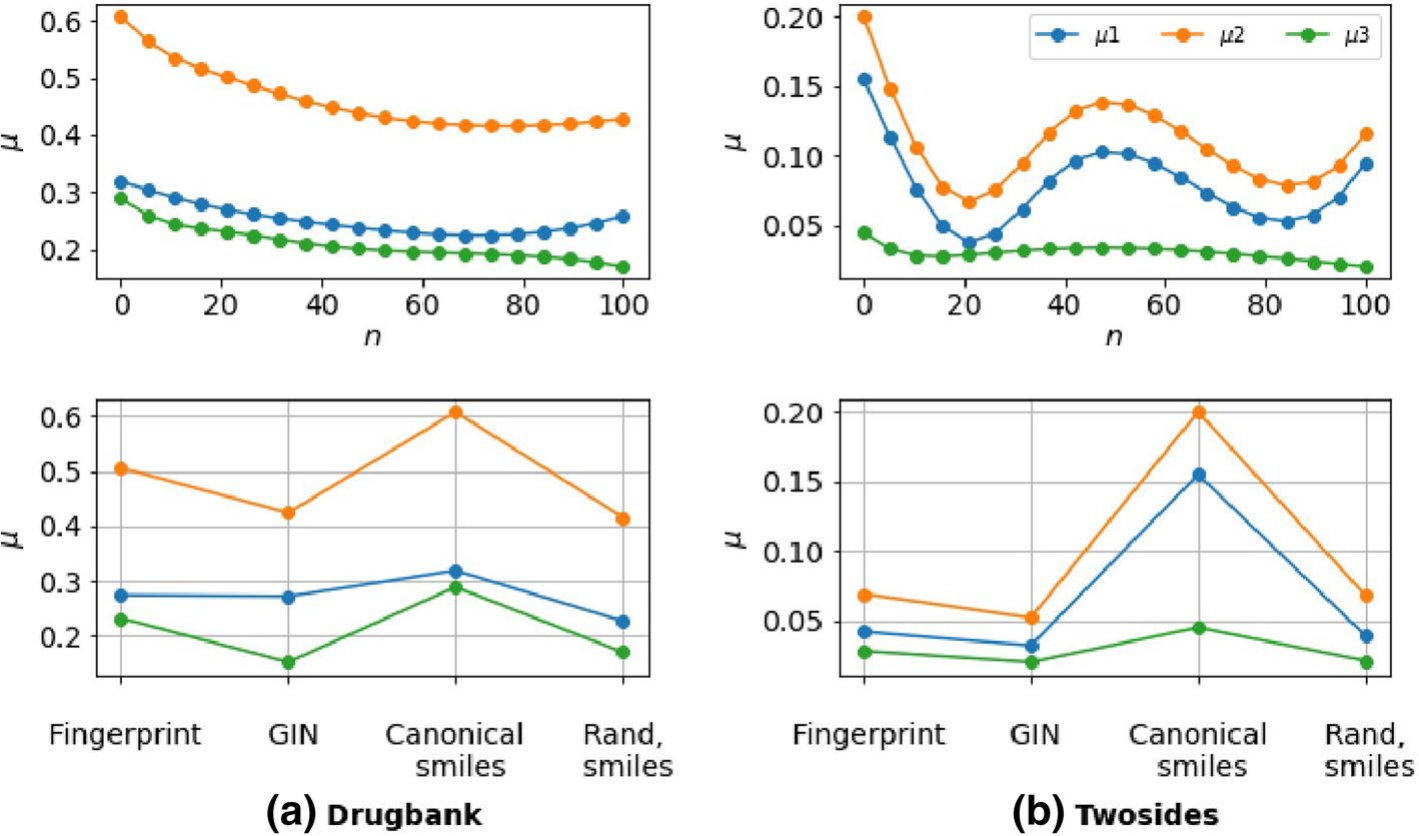
SMILES Data Augmentation results. The first row: We tested different values of n and report the gap between our evaluations schemes. ($\mu 1$ = random–one unseen, $\mu 2$ = random–both unseen, $\mu 3$ = one-unseen–both-unseen). Best n = 20 for Twosides and n = 80 for DrugBank. Second row: we compare ourselves, i.e. best-randomized smiles (Rand, smiles) to GIN, fingerprints, and canonical SMILES

In Fig. 4, we tackled the stability aspect of the models. Figure 4 presents the score for the AUROC and the AUPRC and displays those metrics’ standard deviation over ten random runs. Indeed, the standard deviation offers a measure of the degree of variation or dispersion of the performance of the models between each random run. Figure 4 shows that most of the tested models had slight performance variations (especially for random split settings), indicating that the models were relatively stable. To evaluate the sensitivity of the models, we use a particular advantage of the SMILES representation. Several different SMILES strings can represent a single molecule. All the variants contain the same atoms but read in a separate order. The number of possible SMILES strings depends on the size of the molecule. The longer the molecule, the more variants can be found. In all the experimentations, we have used (as in the literature) the canonical smiles, which is a consensus between the different SMILES string representation for a molecule. To investigate the sensitivity, we generated for each molecule in the training sets a fixed number *n* of SMILES strings using the cheminformatics library RDKit. This technique is known as data augmentation and is widely used to build robust models [52, 53]. We mainly used the 1D-CNN and the GIN models, which respectively register the best performance for random + one-unseen and both-unseen. Figure 6 shows how the performance gap $\mu$ evolves according to the number*n* of randomized smiles generated per drug when switching from the random scheme to one-unseen and both-unseen. We note that using augmented dataset considerably reduced the initial gap between the different evaluation schemes. We also observed that graphs (GIN) and CNN + randomized smiles generated the lowest values compared to fingerprints, with special mention for graphs that continue to have the best performance on both-unseen (Additional file 1: Table S1, Additional file 1: Table S2 and Additional file 1: Table S3). Moreover, the GIN model is more robust and less sensitive than the 1D-CNN model to the different SMILES variations (Additional file 1: Table S2 and Additional file 1: Table S3). This is explained because the molecular graph is the same for canonical SMILES and all its variants.

### Multitask learning: adding an auxiliary task

We attempted to improve the models’ ability to generalize to new unseen molecules using a multitask framework. To do so, we added a new auxiliary task to the learning process. The goal was to learn shared drug representations across the tasks. We assumed that those representations would be helpful to our main task. Hence, the auxiliary task should be related to the main task in some way to share a common optimal hypothesis space, i.e. to have the same inductive bias [54].

As an auxiliary task, we chose the Connectivity Map (CMAP) score prediction task, which predicts for each pair of drugs a score ranging from $-100$ to 100 related to how the two drugs are similar in transcriptional response. Recent findings strengthen the hypothesis that gene-expression changes can, to some extent, reflect drug activities and provide information about mechanisms of action (MoA) [55–57]. It also informs on the molecular targets since high similarity transcriptional responses could represent valid and previously unrecognized connections, e.g. between two proteins operating in the same pathway, between a small molecule and its protein target, or between two small molecules of a similar function but structural dissimilarity. The Connectivity Map (CMap) scores of drug pairs are from the Touchstone dataset (Touchstone V1.0) provided by the Broad Institute [58]. Transcriptional profiles used to compute CMap score in Touchstone are obtained from high-throughput gene expression profiling technology (L1000), which measures the mRNA transcript abundance of 978 “landmark” genes from human cells. The “L” in L1000 refers to the Landmark genes measured in the assay.

For multitask learning, we readapted the training schemes to fit with the new learning task. Therefore, we tried many new protocols to evaluate which ones yielded the best results. First, we co-trained a joint network using: (a) an identical batch for all models during the training phase, i.e. the same drugs pairs samples; then a filter (mask) is used to handle the drugs pairs samples that do not belong to the two tasks (C1), (b) random batches during training; each model has its own drugs pairs samples. Second, we pre-trained our drug feature extractor for CMap prediction and then: (c) freeze the weights of the drug features extractor (T1) and (d) start with the pre-trained model features extractor weights to train a new model (T2). The training scheme that yielded the best results was co-training C1 (see Additional file 1: Table S3). We achieved a maximum improvement of 5% and 2% respectively on the macro and micro performance for the SMILES models when using the random evaluation scheme. Compared to the random scheme, the improvements were slightly less significant for the one-unseen and both-unseen (maximum improvement of 3%) (Additional file 1: Table S4, Additional file 1: Table S5, Additional file 1: Table S6 and Additional file 1: Table S7). No significant improvements were noted for the graph models (Additional file 1: Table S6).

**Fig. 7:**
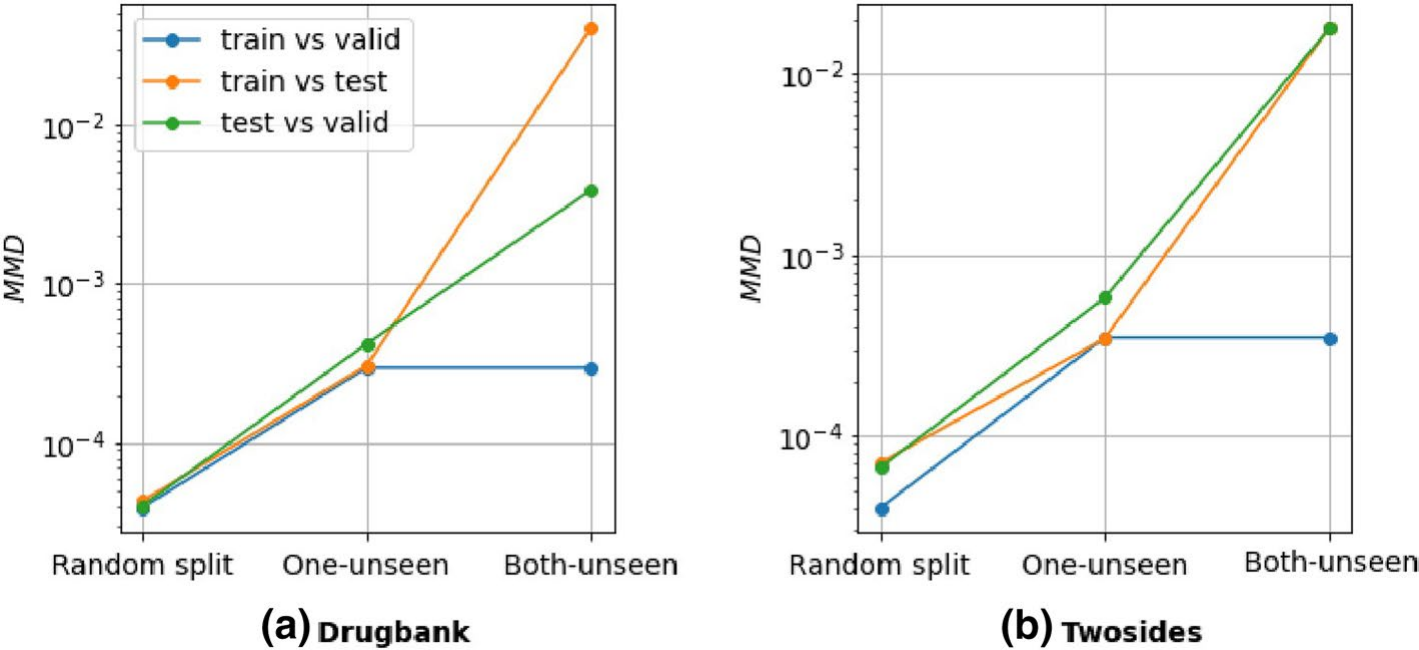
Maximum mean discrepancy (MMD) between drug features distributions. We estimated the maximum mean discrepancy between drug features distributions in train, test, and validation partitions for each of our evaluation schemes

To better understand why multitasking does not seem to work, we quantified the distance between the training, validation, and test sets drugs features distributions via the concept of Maximum Mean Discrepancy (MMD). MMD is defined by the idea of representing distances between distributions as distances between mean embeddings of features. The further away from random, the more the MMD increases (Fig. 7), showing that domain adaptation framework could be more suitable in our specific case. For the prediction of CMap scores, we obtained results similar to the state-of-the-art (MSE = 0.084 [58]).

### Database validation: identifying new drug repositioning candidates

Lastly, we checked the quality of the datasets used by investigating all negative samples labeled 0 in Twosides (ground truth, i.e. TN) for which the output of our best model, the 1D-CNN model, was greater than or equal to 0.7 ($\ge$ 0.7) and confirmed that there is a substantial body of evidence in validated databases and published papers that demonstrate the existence of these interactions. This experiment was inspired by Fig. 5, which shows that model performance degrades as the number of negative samples increases, especially on Twosides. Table 5 shows ten potential DDIs examples with possible side effects, their frequency in the dataset, and the probability output by our model.

We also investigated all drug interactions labeled 1 in Twosides (ground truth, i.e. TP), which obtained very low probabilities ($\le$ 0.1) (our model labeled them 0, i.e. FN). Table 4 shows the ten DDIs examples selected with their potential side effects (as stated in the Twosides database), their frequency in the dataset, and the probability output from our model. False Negative side effects predicted were generally non-intuitive and meaningless (drug withdrawal, adverse drug effect, road traffic accident) or related to sexually transmitted diseases. For example, it was easy to determine that in the case of sexually transmitted diseases, drug combinations can make the disease worse but not cause it. It is also counter-intuitive to think about the drug’s withdrawal as a side effect except for addictive drugs. Nevertheless, it can also be a consequence of a potentially harmful side effect. The above observations are consistent with [59] study, which showed that a small number of novel drug interactions reported in Twosides have been corroborated by thorough investigations of relevant patient records as well as laboratory experiments (Table 5).

**Table 4:** Uncovering the False Negatives examples in Twosides database

Drug1	Drug2	Side effect (SE)	SE freq (%)	Probability		
				Random	One-unseen	Both-unseen
Fenofibrate	Valsartan	Hepatitis c	5.1	0.008	–	–
Verapamil	Fluvoxamine	Hepatitis c	5.1	0.019	0.034	–
Bupropion	Fluoxetine	Hepatitis a	1.0	0.052	0.008	–
Bupropion	Fluoxetine	HIV disease	0.9	0.084	0.009	–
Paroxetine	Risperidone	HIV disease	0.9	0.035	0.006	–
Mupirocin	Sertraline	Drug withdrawal	12.5	0.015	0.107	0.056
Amlodipine	Cerivastatin	Flu	10.4	0.028	0.050	–
Metoclopramide	Minoxidil	Road traffic accident	14.6	0.048	0.005	–
*n*-Acetylcysteine	Cefuroxime	Herpes simplex	6.8	0.029	0.028	–
Clonazepam	Salmeterol	Adverse drug effect	7.5	0.048	–	0.048

The table shows the drug–drug potential side effect and their probability of existence predicted by the 1D-CNN model

**Table 5:** Uncovering potential new DDIs from the 1D-CNN model

Drug1	Drug2	Side effect (SE)	SE freq (%)	Probability			Proof
				Random	One-unseen	Both-unseen	
Bupropion	Benazepril	Heart attack	25.8	0.951	–	0.182	e-1
Didanosine	Stavudine	Acidosis	13.6	0.872	0.068	–	2-2
Bupropion	Fluoxetine	Panic attack	10.5	0.868	0.100	–	e-3
Bupropion	Orphenadrine	Muscle spasm	24.2	0.865	0.161	–	e-4
Tramadol	Zolpidem	Diaphragmatic hernia	1.4	0.854	0.029	0.041	e-5
Paroxetine	Fluticasone	Muscle spasm	24.2	0.871	0.355	–	e-6
Paroxetine	Fluticasone	Fatigue	38.2	0.863	0.450	–	e-7
Fluoxetine	Methadone	Pain	37.6	0.769	–	–	e-8
Carboplatin	Cisplatin	Blood sodium decreased	23.1	0.749	0.097	0.080	e-9
Chlorthalidone	Fluticasone	High blood pressure	30.5	0.797	0.439	–	e-10

The table shows the drug–drug potential side effects supported with the existence probability predicted with the literature link proof

**Fig. 8:**
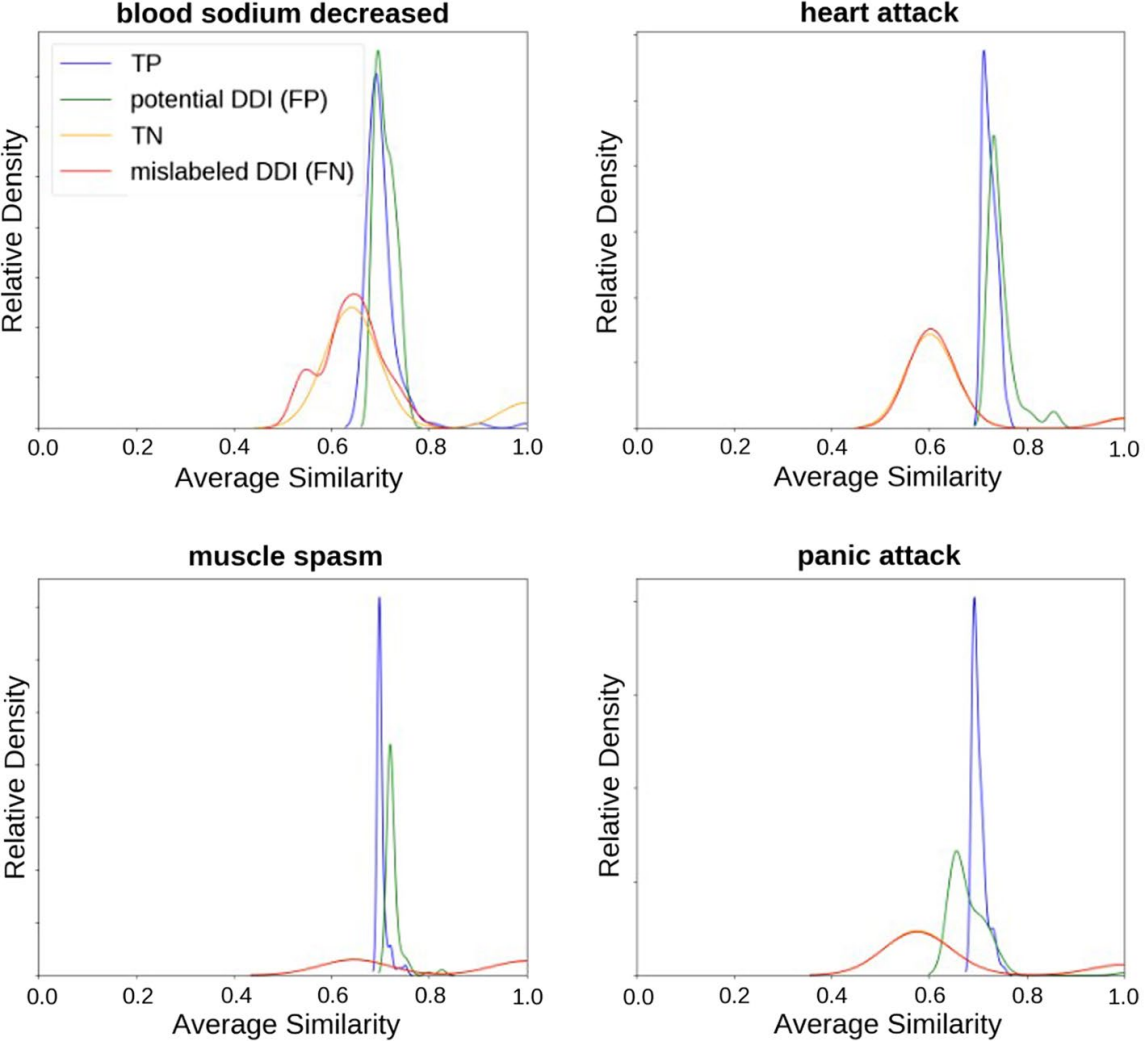
Distribution of similarity of drugs for four candidates side effects. Examples are from Twosides database

Moreover, we confirmed the similarity in the distribution of TP, TN, FN (mislabeling errors), and FP (i.e. potential DDIs) testing pairs compared to positive training samples with the same phenotypes. Figure 8 presents that distribution on 4 different phenotypes. The similarity of two drug pairs (*a*, *b*) and (*c*, *d*) is given by $0.5 \times max(t(a, c), t(a, d)) + 0.5 \times max(t(b, c), t(b, d))$, where *t* is the Tanimoto similarity using ECFP6 fingerprints [60]. On one hand, the similarity distributions of true positives are almost identical to those of false positives, making false positives (FP) more trustworthy candidates for DDIs. On the other hand, the similarity distributions of true negatives are almost identical to those of false negatives, which makes the false negatives (FN) candidates for mislabeling.

## Discussion and conclusion

We investigated and presented an overview of the methods for predicting drug interactions. Most of these methods use the molecular structure of drugs to overcome the limited information available at the beginning of the drug development process for potential new medicines. However, other factors must be considered to improve the models to enhance the drug discovery process.

### Model generalization

The primary factor impacting the ability to generalize existing models to new unseen molecules is the lack of standardization of the training process. We show that current studies use overly optimistic training modes, especially if the goal is to predict interactions between new drugs. Indeed, despite their ability to identify new DDIs amongst drugs seen during training, current model performances decrease significantly if they have to generalize to unseen drugs. Therefore, they can limit the search space of possible drug combinations and provide an alternative starting point towards repurposing old drugs. To predict interactions between new molecules, it is then more realistic to train models using disjointed evaluation schemes similar to both-unseen and one-unseen, while taking into account the distribution of phenotypes in the training set. In addition, the results of “Multitask learning: adding an auxiliary task” section strongly suggest that domain adaptation (DA) (or transfer learning) could improve the prediction of side effects for new drugs.

### Robustness of models

We tested different drug encoding methods for our models: SMILES, molecular graphs, and fingerprints. Each representation is suited for a specific model. The SMILES representation is well suited for the bidirectional long short-term memory model (BLSTM) and the 1D convolutional neural network model (CNN) because BLSTM and 1D-CNN can capture sequential information of string input. The GIN models and their variations are well suited for the molecular graphs’ representation. The deep feedforward neural network is used with fingerprints. Our results demonstrate that recurrent models (BLSTM and CNN) and graph models perform better than feedforward neural networks using fingerprints. They also show that graph models are ideal for predicting interactions between new molecules and are much less sensitive to the order in which the molecule’s components are read. This is not the case for recurrent networks, which are highly dependent on how SMILES are generated. It is therefore recommended to use several variants of the same molecule (SMILES) (“Investigating the robustness of models” section) during training to build robust feature extractors, which will help recurrent models to generalize even better when using the random scheme.

### Database validation

A qualitative analysis of the results (“Database validation: identifying new drug repositioning candidates” section) indicates the need for experimental validation of all the interactions reported in databases such as Twosides, which are derived from data mining. Furthermore, databases should be constructed to provide interactions and non-interactions (true negatives) to help develop reliable prediction algorithms. It is, therefore, preferable to use a curated reference set of drug interactions instead of data obtained through data mining.

## Supplementary Information

**Additional file 1.** Multitask learning and SMILES data augmentation results for 1D-CNN and GIN models.

## Data Availability

All data generated or analyzed during this study are included in this published article. The code is available at: https://github.com/srkpa/drug_drug_interactions/tree/dev_srkpa/side_effects
