# Supplementary material for: On the robustness of generalization of drug–drug interaction models
Source: BMC Bioinformatics. 2021 Oct 4;22:477. doi: 10.1186/s12859-021-04398-9 (PMC8489092; doi:10.1186/s12859-021-04398-9)
Supplement: Supplementary file 1 — Additional file 1. Multitask learning and SMILES data augmentation results for 1D-CNN and GIN models. [file 12859_2021_4398_MOESM1_ESM.pdf]

Multitask learning and SMILES data augmentation results for 1D-CNN and GIN for each evaluation scheme

Table 1: SMILES Data Augmentation on 1D-CNN. We test here different values of n, and reported the gap between our evaluation scheme for each n. Best n = 20 for Twosides and n = 80 for DrugBank. The three rows of the table with the labels graph, fp (fingerprints), and canonical smiles are the benchmarks we compare ourselves to. $\mu 1$ = random -one unseen, $\mu 2$ = random -both unseen, $\mu 3$= one-unseen - both-unseen

Dataset	$\mu 1$	$\mu 2$	$\mu 3$	drug encoding	n
DrugBank	0,274	0,506	0,232	fingerprint	-
	0,272	0,424.	**0,152**	**graph**	-
	0,319	0,608	0,289	canonical, SMILES	-
	0,292	0,538	0,245	Rand, SMILES	10
	0,271	0,504	0,232	Rand, SMILES	20
	0,245	0,452	0,207	Rand, SMILES	40
	**0,227**	**0,416**	**0,189**	**Rand, SMILES**	**80**
	0,258	0,428	0,170	Rand, SMILES	100
twosides	0,042	0,069	0,028	fingerprint	-
	0.032	0,052	0,020	graph	
	0,155	0,200	0,045	canonical, SMILES	–0
	0,080	0,110	0,029	Rand, SMILES	10
	**0,039**	**0,068**	**0,029**	**Rand, SMILES**	**20**
	0,092	0,127	0,034	Rand, SMILES	40
	0,055	0,082	0,028	Rand, SMILES	80
	0,095	0,116	0,021	Rand, SMILES	100

Table 2: SMILES data augmentation on GIN. GIN model was trained using firstly only canonical SMILES, and finally, with randomized SMILES. For each molecule,100 valid randomized SMILES strings were generated.

	SMILES	Evaluation scheme	Model	seed	dataset	AUROC (micro)	AUPRC (macro)
0	Canonical	One-unseen	GIN	42	drugbank	0,969	0,477
1	Randomized	One-unseen	GIN	42	drugbank	0,968	0,468
2	Canonical	Both-unseen	GIN	42	drugbank	0,951	0,324
3	Randomized	Both-unseen	GIN	42	drugbank	0,946	0,309
4	Canonical	One-unseen	GIN	42	twosides	0,790	0,238
5	Randomized	One-unseen	GIN	42	twosides	0,787	0,236
6	Canonical	Both-unseen	GIN	42	twosides	0,775	0,218
7	Randomized	Both-unseen	GIN	42	twosides	0,773	0,216
8	Canonical	random	GIN	42	twosides	0,806	0,270
9	Randomized	random	GIN	42	twosides	0,816	0,286
10	Canonical	random	GIN	42	drugbank	0,993	0,748
11	Randomized	random	GIN	42	drugbank	0,992	0,716

Table 3: SMILES data augmentation on 1D-CNN. 1d-CNN model was trained using firstly only canonical SMILES, and finally, with randomized SMILES. For each molecule,100 valid randomized SMILES strings were generated.

	SMILES	Evaluation scheme	Model	seed	dataset	AUROC (micro)	AUPRC (macro)
0	canonical	One-unseen	CNN	42	twosides	0,800	0,248
1	Randomized	One-unseen	CNN	42	twosides	0,772	0,209
2	canonical	Both-unseen	CNN	42	twosides	0,753	0,203
3	Randomized	Both-unseen	CNN	42	twosides	0,752	0,189
4	canonical	random	CNN	42	twosides	0,870	0,403
5	Randomized	random	CNN	42	twosides	0,823	0,305
6	canonical	random	CNN	42	drugbank	0,999	0,957
7	Randomized	random	CNN	42	drugbank	0,992	0,700
8	canonical	One-unseen	CNN	42	drugbank	0,981	0,634
9	Randomized	One-unseen	CNN	42	drugbank	0,959	0,441
10	canonical	Both-unseen	CNN	42	drugbank	0,922	0,345
11	Randomized	Both-unseen	CNN	42	drugbank	0,927	0,270

Table 4: Multitask learning strategies results. We explore 4 learning strategies to train a joint network for DDIs and CMap scores prediction.

Learning strategy	dataset	evaluation scheme	seed	AUROC (macro)	AUPRC (macro)	AUROC (micro)	AUPRC (micro)
Single task	twosides	Random	42	0.82	0.28	0.87	0.40
C1	twosides	Random	42	0.87	0.34	0.89	0.42
C2	twosides	Random	42	0.68	0.12	0.81	0.25
T1	twosides	Random	42	0.85	0.32	0.88	0.41
T2	twosides	Random	42	0.77	0.23	0.85	0.35

Table 5: C1 on 1D-CNN. Evaluation scheme = one-unseen

Model	dataset	Evaluation scheme	seed	AUROC (macro)	AUPRC (macro)	AUROC (micro)	AUPRC (micro)
Single task	twosides	one-unseen	42	0.676	0.117	0.800	0.248
C1 No Regularization	twosides	one-unseen	42	0.728	0.139	0.817	0.257
C1 dropout = 0.2	twosides	one-unseen	42	0.733	0.142	0.822	0.262
C1 dropout = 0.4	twosides	one-unseen	42	0.729	0.139	0.822	0.268
C1 dropout = 0.8	twosides	one-unseen	42	0.694	0.126	0.807	0.255
C1 weight decay = 1e-05	twosides	one-unseen	42	0.734	0,146	0.817	0.260
C1 weight decay =1e-04	twosides	one-unseen	42	0.682	0.135	0,796	0.257
C1 weight decay=1e-3	twosides	one-unseen	42	0.611	0.104	0.738	0.193
C1 weight decay=1e-02	twosides	one-unseen	42	0.572	0,089	0.63	0.099

Table 6: C1 on 1D-CNN. Evaluation scheme = both-unseen

Model	dataset	Evaluation scheme	seed	AUROC (macro)	AUPRC (macro)	AUROC (micro)	AUPRC (micro)
Single task	twosides	both-unseen	42	0.582	0.102	0.753	0.203
C1 No Regularization	twosides	both-unseen	42	0.545	0.095	0.754	0.200
C1 dropout = 0.2	twosides	both-unseen	42	0.562	0.103	0.770	0.215
C1 dropout = 0.4	twosides	both-unseen	42	0.560	0.099	0.773	0.215
C1 dropout = 0.8	twosides	both-unseen	42	0.569	0.101	0.776	0.226
C1 weight decay = 1e-05	twosides	both-unseen	42	0,551	0.102	0.74	0.198
C1 weight decay =1e-04	twosides	both-unseen	42	0.546	0.111	0.692	0.172
C1 weight decay=1e-3	twosides	both-unseen	42	0.533	0.098	0.64	0.108
C1 weight decay=1e-02	twosides	both-unseen	42	0.54	0.094	0.548	0.075

Table 7: C1 on GIN. Evaluation scheme = One-unseen & both-unseen

dataset	seed	model	Evaluation scheme	AUROC (micro)	AUPRC (micro)	AUROC (macro)	AUPRC (macro)
twosides	42	GIN	Both-unsen	0.775	0.218	0.551	0.100
twosides + Touchstone	42	GIN + C1	Both-unseen	0.785	0.23	0.58	0.105
twosides	42	GIN	One -unseen	0.800	0.240	0.600	0.101
twosides + Touchstone	42	GIN + C1	One-unseen	0.794	0.245	0.629	0.108

Table 8: Maximum Mean Discrepancy (MMD) between drug features distributions. For each of our evaluation scheme, we estimate the maximum mean discrepancy for drug features distributions between partitions.

Dataset	Evaluation scheme	train/valid	train/test	test/valid
Twosides	Random	4e-05	7,1e-05	6,7e-05
	One-unseen	3,49e-04	3,45e-04	5,79e-04
	Both-unseen	3,49e-04	1,8e-02	1,78e-02
DrugBank	Random	3,9e-05	4,3e-05	4e-05
	One-unseen	2,97e-04	3,07e-04	4,1e-04
	Both-unseen	2,97e-04	4,108e-02	3,9e-03
